# Facts and Perspectives: Implications of tumor glycolysis on immunotherapy response in triple negative breast cancer

**DOI:** 10.3389/fonc.2022.1061789

**Published:** 2023-01-10

**Authors:** Ashley Schreier, Roberta Zappasodi, Inna Serganova, Kristy A. Brown, Sandra Demaria, Eleni Andreopoulou

**Affiliations:** ^1^ Division of Hematology and Medical Oncology, Department of Medicine, Weill Cornell Medicine, New York Presbyterian Hospital, New York, NY, United States; ^2^ Division of Hematology and Medical Oncology, Department of Medicine, Weill Cornell Medicine, New York, NY, United States; ^3^ Immunology and Microbial Pathogenesis Program, Weill Cornell Graduate School of Medical Sciences, New York, NY, United States; ^4^ Parker Institute for Cancer Immunotherapy, San Francisco, CA, United States; ^5^ Human Oncology & Pathogenesis Program, Memorial Sloan Kettering Cancer Center, New York, NY, United States; ^6^ Department of Medicine, Weill Cornell Medicine, New York, NY, United States; ^7^ Department of Radiation Oncology and Department of Pathology, Weill Cornell Medicine, New York, NY, United States

**Keywords:** triple negative breast cancer (TNBC), immune microenviroment, tumor glycolysis, regulatory T (Treg) cell, immune checkpoint inhibitor (ICI)

## Abstract

Triple negative breast cancer (TNBC) is an aggressive disease that is difficult to treat and portends a poor prognosis in many patients. Recent efforts to implement immune checkpoint inhibitors into the treatment landscape of TNBC have led to improved outcomes in a subset of patients both in the early stage and metastatic settings. However, a large portion of patients with TNBC remain resistant to immune checkpoint inhibitors and have limited treatment options beyond cytotoxic chemotherapy. The interplay between the anti-tumor immune response and tumor metabolism contributes to immunotherapy response in the preclinical setting, and likely in the clinical setting as well. Specifically, tumor glycolysis and lactate production influence the tumor immune microenvironment through creation of metabolic competition with infiltrating immune cells, which impacts response to immune checkpoint blockade. In this review, we will focus on how glucose metabolism within TNBC tumors influences the response to immune checkpoint blockade and potential ways of harnessing this information to improve clinical outcomes.

## Introduction

Recent clinical advances using immunotherapy have provided new treatment possibilities for women who have triple-negative breast cancer (TNBC), one of the most challenging subtypes of breast cancer to treat. TNBC lacks expression of estrogen receptor (ER), progesterone receptor (PR) or HER2/neu gene amplification, and accounts for 11-15% of breast cancers in the US. Compared to ER-positive breast cancer, TNBC is associated with a higher risk of distant recurrence, higher rates of visceral and central nervous system metastases, and earlier time to recurrence (1–3). The current treatment of this aggressive disease is mainly limited to the use of cytotoxic chemotherapy. Unlike in ER-positive and HER2-positive breast cancers where tremendous improvements in clinical outcomes have been realized through the use of targeted agents, there are currently no approved targeted treatments for TNBC, other than PARP inhibitors in the small subset of patients with *BRCA1* or *BRCA2* mutant tumors. Genotyping studies have enabled the identification of distinct molecular subtypes of TNBC which has elucidated some of the heterogeneity of these tumors (3–7). However, clinical use of this information to guide precision-medicine treatment strategies beyond the standard of care is still evolving. The aggressive biology along with the paucity of treatment options for TNBCs make this disease difficult to treat, and patients are often faced with a grim prognosis, particularly in the metastatic setting. Even in early stages, TNBC portends a very poor prognosis in certain patients. Importantly, it is now clear that a sizable subset of TNBCs are immunogenic and can respond to immune checkpoint inhibitors (ICIs) (8, 9). Landmark trials have led to the FDA approval of PD-1 inhibitors in TNBC, which have become part of the standard of care in PD-L1 positive metastatic patients and more recently in the neoadjuvant and adjuvant setting, regardless of PD-L1 status (10, 11).

Immunotherapy addresses a crucial component of cancer’s ability to thrive and proliferate - the escape from immune surveillance. ICIs have created a shift in the TNBC treatment paradigm, by offering an opportunity for remarkable and durable outcomes in a subset of patients. The introduction of these therapies has been particularly exciting in early-stage disease, where curative intent is paramount. Neoadjuvant treatment has been the preferred management path in early-stage TNBC, with achievement of a pathologic complete response (pCR) established as a prognostic factor; and patients with residual disease at the time of surgery having up to a 75% chance of recurrence within 10 years (12–15). Neoadjuvant immune checkpoint blockade has significantly improved this outcome, but suboptimal patient selection still limits the breadth of its benefit. Recent research efforts have focused on discovering the mechanisms underpinning the lack of response to immunotherapy seen in many patients, aiming to identify actionable targets in order to re-program immune-resistant tumors for a favorable response.

The unfortunate outcome of the roughly 50% of TNBC patients with residual disease after neoadjuvant treatment indicates the crucial need to optimize primary treatment strategies for early-stage disease, where cure is the ultimate goal. An interesting approach towards treatment optimization is one that targets multiple hallmarks of cancer and their interactions. Among them, “*avoiding immune destruction*” and “*deregulating cellular energetics*” play crucial roles in tumor growth both independently and in relation to one another, as conceptualized by Hanahan and Weinberg in 2011 in their revised framework of “hallmarks of cancer” (16). Preclinical studies have shown that highly metabolic triple negative mammary tumors, particularly those with increased aerobic glycolysis, have limited responsiveness to immunotherapy (17–19). The metabolically harsh microenvironment posed by highly glycolytic tumors and its negative impact on anti-tumor immune responses may offer an opportunity for therapeutic intervention and for rational combination treatments that favor tumor response to immunotherapy. Moreover, the potential for improved immune responses in glycolysis-low tumors invites a new strategy to select patients who may benefit the most from immunotherapy, and those in whom tumors’ metabolic dependencies can be reprogrammed to favor a response. In this article, we review the current stage of clinical development of ICIs in TNBC and discuss recent studies exploring the associations between tumor glycolysis and anti-tumor immunity in the setting of ICIs and the implications for these mechanisms in the treatment of TNBC.

## Clinical development of immune checkpoint inhibitors for TNBC 

The advent of ICIs – with the anti-CTLA-4 antibody ipilimumab initially approved for use in metastatic melanoma in 2011, followed by the approval of anti-PD-1 antibodies nivolumab and pembrolizumab in 2014 – has revolutionized the treatment of a variety of solid tumors. However, the success in breast cancer has lagged behind. This has been attributed to the immunosuppressive tumor microenvironment (TME) traditionally described in breast cancer across subtypes and to the overall lower immunogenicity of these tumors – including poorer lymphocyte infiltration and decreased tumor mutational burden – compared to immune responsive solid tumors, such as melanoma and lung cancer (20). More recently, distinctive immune traits have been described for TNBC, pointing to this disease as the most immunogenic subtype of breast cancer. Studies have shown increased immune cell infiltration in TNBC tumors compared to other breast cancer subtypes, and elevated frequencies of tumor infiltrating lymphocytes (TILs) have been associated with improved survival in TNBC (8, 21–25). This can explain the superior response to immune checkpoint blockade observed in a subset of TNBC patients. Several clinical trials continue to explore various treatment combinations with immune-based therapeutics with the goal of improving clinical outcomes for TNBC patients. The use of immunotherapy in triple negative breast cancer has been extensively reviewed previously and will be summarized briefly here (26–29).

The current standard of care for ICI use in TNBC was set by several landmark trials, which are highlighted in **Table 1**. Initial studies using single agent anti-PD1 and anti-PD-L1 antibodies showed a clinical benefit in very few patients with metastatic disease, with responses being most prominent in the first line setting, and in those with PD-L1 positive tumors. Subsequent trials in selected patient populations with advanced PD-L1-positive (PD-L1+) TNBC led to the initial FDA approval of the PD-L1 inhibitor atezolizumab in this setting. This was ultimately achieved based on results from the phase III Impassion130 trial that tested atezolizumab in combination with nab-paclitaxel as first -line treatment in metastatic TNBC patients. This trial randomized 902 patients with advanced TNBC to nab-paclitaxel with the addition of either atezolizumab or placebo. A progression free survival (PFS) of 7.2 months was seen in the atezolizumab compared to 5.5 months in the placebo group (HR= 0.80; 95% confidence interval [CI], 0.69 to 0.92; P=0.002) (33). However, in the Impassion131 trial, the combination of atezolizumab with paclitaxel did not show clinical benefit in a similar patient population (34), leading to a halt of the FDA’s accelerated approval of atezolizumab for metastatic TNBC until additional data matures (39). Steroid use with paclitaxel may be one explanation for the discrepancy in the results between these two trials.

**Table 1:** Clinical trials of immune checkpoint inhibitors in breast cancer.

Trial	Treatment	ICITarget	Phase	Setting/Line of therapy	N for TNBC	Results in patients with TNBC
**JAVELIN** (30)	Avelumab single agent	PD-L1	Ib	Metastatic Heavily pre-treated; Median of 3 prior lines for metastatic disease	58	ORR of 5.2%; trend towards higher ORR in PD-L1+ tumors (22.2% vs. 2.6%)
**KEYNOTE- 119** (31)	Pembrolizumab single agent vs. single agent chemotherapy per investigator’s choice	PD-1	III	Metastatic 2^nd^ or 3^rd^ line	622	CPS≥10: mOS of 12.7 vs 11.6 mo (HR 0.78, p=0.057) CPS ≥ 1: mOS of 10.7 vs 10.2 mo (HR 0.86, p= 0.07) Overall: mOS 9.9 vs 10.8 mo (HR 0.97)
**ENHANCE-1** (32)	Eribulin plus pembrolizumab	PD-1	Ib/II	Metastatic 1^st^ – 3^rd^ line	167	ORR in 1^st^ line: 25.8% ORR in 2^nd^-3^rd^ line: 21.8% ORR in CPS ≥ 1 and 1^st^ line: 34.5% vs. 16.1% in PD-L1 negative tumors ORR in CPS ≥ 1 and 2^nd-3rd^ line: 24.4% vs. 18.2% in PD-L1 negative tumors
**IMPASSION-130** (33)	Nab-paclitaxel +/- Atezolizumab	PD-L1	III	Metastatic 1^st^ line	902	mPFS of 7.2 vs. 5.5 mo (HR 0.80, P= 0.002) PD-L1 positive tumors: mPFS 7.5 vs. 5.0 mo (HR 0.62, p < 0.001) Median OS of 21.3 vs. 17.6 mo (HR 0.84, p=0.08) PD-L1 positive tumors: mOS of 25 vs. 15.5 mo (HR 0.62)
**IMPASSION-131** (34)	Paclitaxel +/- Atezolizumab	PD-L1	III	Metastatic 1^st^ line	651	mPFS in patients with PD-L1 positive tumors: 6.0 vs. 5.7 mo (HR 0.82, p=0.2) ORR in PD-L1 positive patients: 63% vs. 55% and median duration of response: 7.2 vs. 5.5 mo OS in patients with PD-L1 positive tumors: 22.1 vs 28.3 mo (HR 1.11)
KEYNOTE-355* (11)	Chemotherapy with taxane or gemcitabine/carboplatin +/- pembrolizumab	PD-1	III	Metastatic 1^st^ line	847	CPS≥10: mPFS of 9.7 vs. 5.6 mo (HR 0.65, p=.0012) CPS ≥ 1: mPFS of 7.6 vs. 5.6 mo (HR 0.74, p=0.0014) ITT: mPFS of 7.5 vs. 5.6 mo (HR 0.82)
**I-SPY2** (35)	Taxane and anthracycline based chemotherapy +/- pembrolizumab	PD-1	II	Neoadjuvant	29	Improved pCR rate to 60% compared to 22% in TNBC patients
**KEYNOTE-173** (36)	Pembrolizumab plus chemotherapy (6 chemotherapy arms)	PD-1	Ib	Neoadjuvant	60	Overall pCR rate of 60% pCR rate of 64% in those with CPS>1 vs. 40% in PD-L1 neg patients pCR of 72% in those with a CPS>30
**Impassion-031** (37)	Chemotherapy +/-atezolizumab	PD-L1	III	Neoadjuvant	333	pCR rate of 58% vs 41% in the atezolizumab compared to placebo group; p=0.0044 pCR rate improved to 69% vs. 49% in the PD-L1 positive patients treated with atezolizumab; p=0.021
KEYNOTE-522* (36)	Chemotherapy +/- pembrolizumab	PD-1	III	Neoadjuvant/adjuvant	1174	Improved pCR rates when pembrolizumab was added to NAC: 64.5% vs. 51.2% (p< 0.001) Improved EFS at a median follow up of 36 months for the pembrolizumab group: 84.5% vs. 76.8% (HR 0.63, P <0.001) Favorable trend in OS in the pembrolizumab group was also observed (HR 0.72, 95% CI 0.51-1.02; P=0.03)
**GeparNEUVO** (38)	Chemotherapy +/-durvalumab	PD-L1	II	Neoadjuvant	174	pCR rate of 53.4% was seen in the durvalumab group compared to 44.2% in the placebo group After a median follow up of 42.2 months, 3-year invasive DFS rate of 84.9% with durvalumab compared to 76.9% with placebo (HR 0.54, p = 0.0559)

*These studies led to current FDA approved indications for ICI use in breast cancer.

In November 2020, the second FDA approval for immunotherapy in breast cancer was granted for pembrolizumab in combination with chemotherapy for the treatment of PD-L1+ metastatic TNBC. This approval was based on results of the phase III KEYNOTE-355 study which randomized 847 patients to chemotherapy with either pembrolizumab or placebo, for previously untreated locally recurrent inoperable or metastatic TNBC. Chemotherapy was either with a taxane or gemcitabine/carboplatin (11). Patients were stratified based on combined positive score (CPS), a scoring system for standardization of PD-L1 protein expression, defined as the ratio of the number of all PD-L1 -expressing cells (tumor cells and immune cells) to the total number of all viable tumor cells; with a maximum score of 100 (40). Median PFS was 9.7 vs. 5.6 months (HR 0.65, 95% CI 0.49 - 0.86) in patients with a CPS score of 10 or greater. In patients with a CPS score of 1 or greater, PFS was 7.6 months vs. 5.6 months (HR 0.74, 95% CI 0.61 - 0.90; p=0.0014), however, there was no difference in PFS in patients with CPS <1. The benefits of pembrolizumab were maintained across different chemotherapy subgroups (11).

Subsequently, ICIs began to be explored in earlier stages of breast cancer and have recently been approved for use in TNBC in the neoadjuvant setting, with continuation for 9 cycles in the adjuvant setting. Fortunately, the clinical outcomes in neoadjuvant and adjuvant ICI trials in TNBC have been more favorable than those seen in the metastatic setting. A pCR rate of 50% has been traditionally reported with anthracycline and taxane based neoadjuvant chemotherapy (NAC) for TNBC, but this figure has improved further with the addition of immunotherapy, particularly with the PD-1 inhibitor pembrolizumab (10, 15, 41, 42). Results from the practice changing KEYNOTE-522 trial showed improved pCR rates when pembrolizumab was added to NAC (64.5% vs. 51.2%, p< 0.001) (10), leading to its FDA approval in this setting in July 2021 (10, 35, 42, 43). Event free survival (EFS) was assessed at a median follow up of 36 months, which showed a meaningful improvement in the pembrolizumab group (HR 0.63, 95% CI 0.48 – 0.82; P<0.001), and this benefit was similar between PD-L1 positive and PD-L1 negative patients. A favorable trend in OS in the pembrolizumab group was also observed (HR 0.72, 95% CI 0.51-1.02; P=0.03) (42). The GeparNEUVO trial evaluated the addition of neoadjuvant durvalumab (anti-PD-L1) to chemotherapy in TNBC; a pCR rate of 53.4% was seen in the durvalumab group compared to 44.2% in the placebo group (44). An updated analysis after a median follow up of 42.2 months of this trial showed improved 3-year invasive disease-free survival (DFS) in the pCR compared to the non-pCR group (92% vs. 72%; p=0.002; with a 3-year invasive DFS rate of 84.9% with durvalumab compared to 76.9% with placebo; HR 0.54, 95% CI 0.27-1.09; p = 0.0559) (38).

Studies have revealed that metastatic breast tumors are much less immunogenic, and are rather immune depleted, compared to primary breast tumors (21, 45–49). It can be postulated that in order to proliferate and metastasize, tumors must escape from immune surveillance and develop resistance mechanisms to immune-based therapies (50). In line with this conclusion, immunotherapy has shown more success in the neoadjuvant and adjuvant settings compared to its use in advanced disease, making early-stage clinical settings the ideal space for immunotherapy implementation and optimization. The neoadjuvant/adjuvant settings present a window of opportunity for cure, and treatment optimization in this space is integral to long term benefit. The exciting role of immunotherapy in the treatment paradigm of triple negative disease has been highlighted by the above data. As the recent FDA approvals for ICIs in TNBC are being incorporated into standard practice, there is an ongoing need to further optimize their use and select patients who may benefit the most. Combination strategies are being explored to take advantage of different drivers of tumor growth and immune escape. A focus on targeting tumor metabolism, especially glycolysis, may be one such strategy to counteract tumor immune evasion and promote the response of TNBC to immunotherapy, as discussed below.

## Characterization of TNBC metabolism and the immune microenvironment

### Distinctive features of TNBC metabolism

Metabolic pathways with important roles in maintaining cellular energy homeostasis are often dysregulated in cancer. Unlike normal cells, cancer cells acquire the ability to easily adapt and reprogram their metabolism in order to sustain anabolic processes, proliferation and tumor progression. A major mechanism of such metabolic reprogramming is the diversion of energy metabolism towards glycolysis, independent of oxygen availability; this is referred to as the Warburg effect (51, 52). Cancer cells increase expression of glucose transporters and glycolytic enzymes, which leads to increased glucose consumption and utilization of glycolysis intermediates to sustain crucial biosynthetic pathways for growth and division. The final glycolytic product – lactic acid – is subsequently excreted into the extracellular space contributing to an acidic and hostile microenvironment for surrounding normal cells (16, 51, 53).

Research efforts have focused on discovering how and why certain cancer cells shift their energy production towards glycolysis more likely than others (53). TNBC tumors in particular have demonstrated more predominantly dysregulated metabolism with a greater reliance on glycolysis compared to non-triple negative breast cancer subtypes, as demonstrated by their upregulation of glucose/lactate transporters and glycolytic enzymes, as well as their distinctive expression of metabolic genes (19, 54–58). Glucose transporters (GLUTs) have been implicated in cancer growth and progression by facilitating influx of glucose into cancer cells for maintenance of biosynthetic processes (59). Further, when treated with anti-GLUT-1 antibodies, breast cancer cell lines have demonstrated increased apoptosis and reduced proliferation, which has also been shown to work synergistically with chemotherapy in preclinical models (60, 61). Hussein et al. showed that increased GLUT-1 expression evaluated by immunohistochemical analysis was correlated with high histologic grade, ER/PR negative tumors, EGFR expression, and high p53 expression (58). In addition, studies have shown that there is significant heterogeneity in the metabolic dependencies and preferences between TNBC tumors (19, 55, 62). For example, Lanning et al. quantified the metabolic profiles of TNBC cell lines and revealed significant metabolic heterogeneity in terms of the metabolic rates and responses to metabolic pathway inhibitors (62). Certain unique metabolic characteristics of the TNBC microenvironment were demonstrated by Ghergurovich et al. in a study where TNBC patients were infused with a glucose isotype tracer prior to breast biopsy. Tumor tissue was then examined for isotype tracing to explore intra-tumoral glucose catabolism and anabolism and this was compared with data from other tumor types. These TNBC tumors demonstrated a preference for glycolysis over oxidative phosphorylation, produced amino acids *de novo* to support protein synthesis and lipid and nucleic acid production, and generated their own lactate rather than acquiring it from circulation (63).The authors concluded that the metabolic microenvironment surrounding TNBC tumors is distinct compared to other tumor types (63). 

A more straightforward and clinically applicable approach to assessing tumor metabolism and glycolytic preferences can be achieved through the use fluorodeoxyglucose (FDG)-positron emission tomography (PET) imaging. Maximum value of FDG uptake in the tumor (SUV_max_) has traditionally been used as a surrogate indicator of glycolytic phenotype in various cancers and is the most commonly reported and best-known PET scan parameter. Other measures such as MTV (metabolic tumor burden) and TLG (total lesion glycolysis; measuring glucose uptake within a region of interest), which are volumetric measures of tumor glycolysis, have been studied as potential biomarkers of response to treatment (64, 65). Parameters derived from PET/CT scans have the potential to predict aggressiveness of disease and clinical outcomes in breast cancer. The change in SUV_max_ during neoadjuvant chemotherapy treatment has been shown to predict pCR in TNBC patients (66, 67), and higher values of SUV, MTV, and TLG were shown to be associated with a higher probability of having axillary metastases in breast cancer patients (68). In the metastatic setting, MTV has been shown to be associated with survival in TNBC patients (69).

Further, PET/CT scans show distinct profiles for different subtypes of breast cancer, highlighting the potential of imaging techniques to resolve tumor metabolism heterogeneity. A study by Basu et al. compared the FDG uptake, measured by SUV_max_, in TNBC versus ER-positive/PR-positive/HER2-negative breast cancer in newly diagnosed patients. The authors found significantly higher SUV_max_ values in the total population of TNBC patients and across subgroups by tumor size, grade, and stage, compared to the ER-positive/PR-positive/HER2-negative breast cancer group, suggesting increased tumor glycolysis may contribute to the aggressiveness of TNBC (70). Similarly, a retrospective study by Kwon et al. studied pre-operative PET/CT scans of 284 newly diagnosed breast cancer patients (36 with TNBC) to correlate PET findings with histopathology, and found triple negative tumors to have significantly higher SUV values than other subtypes (71). Accordingly, in a prior prospective study by Garcia Vicente et al. in 168 patients, SUV values in PET/CT scans obtained before neoadjuvant treatment correlated with molecular breast cancer subtype, with significantly higher SUV measures seen in HER2-positive tumors and in the basal-like subtype, which is mostly comprised of TNBC (72). High TLG and MTV have also shown to correlate with the basal-like molecular subtype of breast cancer (73). A better understanding of tumor metabolism and glycolytic preferences in TNBC has the potential to inform clinical studies assessing the utility of these metabolic parameters in practice, and how they may serve a role in predicting outcome to certain therapies, including immunotherapy.

Initial work to this aim has shown that it is possible to measure the effects of metabolic interventions using FDG PET/CT, including the unique challenges to consider when interpreting this in the context of immunotherapy. In a TNBC xenograft model, Miao et al. used a chemically-synthesized microRNA (miR-143) to inhibit glycolysis through the downregulation of hexokinase 2 (HK2), which resulted in reduction of tumor growth and corresponding decreases in FDG uptake by PET/CT (74). These data suggest the possibility of using FDG PET/CT scans to assess glycolysis within TNBC tumors, as decreased rates of glucose consumption and lactate production through HK2 targeting correlated with reduced FDG uptake (74). However, PET scan parameters may also be influenced by the tumor’s size and microenvironment, especially when actively proliferating lymphocytes infiltrate the tumor, which usually correlates with better prognosis. The inability to distinguish between FDG uptake by glycolytic tumor cells and reactive lymphocytes mounting robust anti-tumor immune responses creates a challenge in the use of PET scans to predict immunotherapy responses. For example, Lopci et al. evaluated the correlation between PET/CT markers and immune-related tissue markers in 55 patients with NSCLC and found a statistically significant correlation between SUV_max_ and the presence of CD8+ TILs (75). A subsequent study with 27 NSCLC patients showed that “fast progressors” on immunotherapy had lower SUV values on baseline PET/CT (76). The interpretation of PET results in the context of immunotherapy may be improved by taking into account the timing of these metabolic assessments with respect to treatment and results from concurrent analyses of the tumor immune composition. These factors will be crucial for understanding the utility of FDG PET/CT scan parameters as predictive biomarkers of immunotherapy response.

### Distinctive features of the TNBC immune microenvironment

Selective pressure from the immune system plays an integral role in shaping malignant transformation, growth and spread of cancer cells, and their resistance to immune responses (77). This has been best summarized through the concept of “cancer immunoediting”, which depicts the relationship between tumor cells and immune cells and their evolution through three distinct sequential phases: elimination, equilibrium, and escape (78). The first phase, elimination, describes the recognition and elimination of tumor cells by T cells, thereby preventing tumor formation. After an equilibrium phase between anti-tumor immune attack and cancer growth, the immune escape phase may eventually prevail, meaning that the immune system fails to restrict the growth of tumor cells and the malignancy manifests (50). This framework elucidates the key role of the immune system in eliminating incipient tumors while shaping the characteristics of the tumors that emerge by exerting a selective pressure (79). It is now clear that in order to progress, tumors have to escape immune-mediated control. Accordingly, persistent tumor infiltration with anti-tumor T cells is associated with a better survival in several cancer types, including TNBC (8, 80). Uncovering the mechanisms of immune escape is fundamental to decipher why certain tumors evade immune control, while others are sensitive to immune attack and how the latter ones can convert into the formers over the course of treatment, thus becoming resistant (81). Such complex cross-talk between the cancer cells and the immune system likely contribute to the heterogeneity of clinical responses observed after treatment with immunotherapy, including ICIs (82), as well as other non-immune based treatments such as chemotherapy (25). The molecular interplay between cancer cells, infiltrating immune cells, and the stroma may be at the core to understanding these resistance mechanisms in many types of cancer, including TNBC (83).

Immunologic parameters such as TILs and immune-related gene signatures in TNBC, HER-2-positive, and high-risk ER-positive breast cancers have all been associated with favorable clinical outcomes (21, 46, 84, 85). Studies have consistently shown that TNBC has the most immune-enriched infiltrate compared with other breast cancer subtypes (21), in line with the increased responsiveness of TNBC to immunotherapy (86–88). In TNBC, the degree of tumor infiltration by lymphocytes has been shown to have both prognostic and predictive value for response to neoadjuvant chemotherapy and ICIs (24, 86–90), and has also shown predictive value in ICI use in patients with metastatic TNBC (91–93). Further, studies have shown that the presence of TILs is an independent prognostic factor of long-term responses in TNBC patients receiving adjuvant chemotherapy (23, 90, 94). It has also been shown that an elevated number of TILs in residual disease after neoadjuvant chemotherapy in TNBC patients who do not achieve pCR is associated with improved long-term prognosis (95).

Gene expression profiling has allowed scientists to classify TNBC into distinct molecular subtypes. The most recent refinements of these analyses have led to the description of 4 subtypes: basal-like 1, basal-like 2, mesenchymal, and luminal androgen receptor (BL1, BL2, M and LAR) (4, 5). Studies have also suggested distinct immunologic characteristics amongst breast cancer subtypes, as well as heterogeneity in the immunogenicity of different TNBCs (24). Gruosso et al. studied a cohort of 38 treatment-naïve TNBC patients to define subgroups according to the presence and localization of CD8+ T cells as well as gene expression profiling to identify biological pathways associated with specific CD8+ T cell localization patterns. TNBC tumors with high CD8+ lymphocytic infiltration showed greater expression of PD-L1 and more favorable outcomes compared to “immune-cold” TNBC tumors, which lacked CD8+ T lymphocyte infiltration. These “immune-cold” tumors were characterized by a more immunosuppressive microenvironment and a more fibrotic stroma, with CD8+ T cells restricted to the tumor margins or stroma (83). Bareche et al. assessed 1512 TNBC samples to characterize the TME amongst the 4 TNBC molecular subtypes and demonstrated that each subtype of TNBC was composed of unique TME features with regards to composition and localization of their immune infiltrate (96). Single cell RNA sequencing of immune cells from breast tumors highlighted the great diversity in immune cell subsets between different breast tumors and noted T cells to exist on a continuum of differentiation/activation states (82). Wagner et al. used mass cytometry profiling of 144 prospectively collected tumor samples to characterize the tumor immune phenotype and found ER-negative breast tumors to have a higher frequency of regulatory T cells (Tregs) compared to ER-positive tumors (97).

Given the role of T cells in inhibiting tumor growth, it is not surprising that tumors that progress to the metastatic stage usually have a low T cell infiltration compared with that of primary breast tumors. Lower TIL counts are seen in the metastatic TME, where PD-L1 expression is also generally decreased compared to that of primary breast tumors (46, 47, 49). Such differences may be even more pronounced in TNBC (48). Szekely et al. studied paired tissue blocks of primary and metastatic breast cancers and reported a decreased expression of immune related genes in the metastatic compared to primary tumors, including those infiltrated by CD8 T cells, activated T cells, and dendritic cells, and also noted lower expression of activated T-cell transcription factors and IFNγ regulated genes in the metastatic TME (46).

These data highlight the complexity of the TNBC immune microenvironment and shed light on how its diverse and dynamic nature poses challenges for defining resistance mechanisms and robust predictive biomarkers of response to immunotherapy, including ICIs. A deepened understanding of the cross-talk between immune cells and TNBC cells is needed to identify key targets of immune-inhibitory mechanisms within the TME for optimized combination treatments. In this regard, emerging studies are pointing to vicious metabolic interactions in the TME that not only promote tumor-intrinsic metabolic fitness but also contribute to suppressing anti-tumor immune responses and to immunotherapy resistance. We discuss below how we can harness these mechanisms to develop more effective and personalized immunotherapy approaches for TNBC.

## Opportunities to optimize treatment

### Tumor glycolysis and immunotherapy response

**Figure 1:**
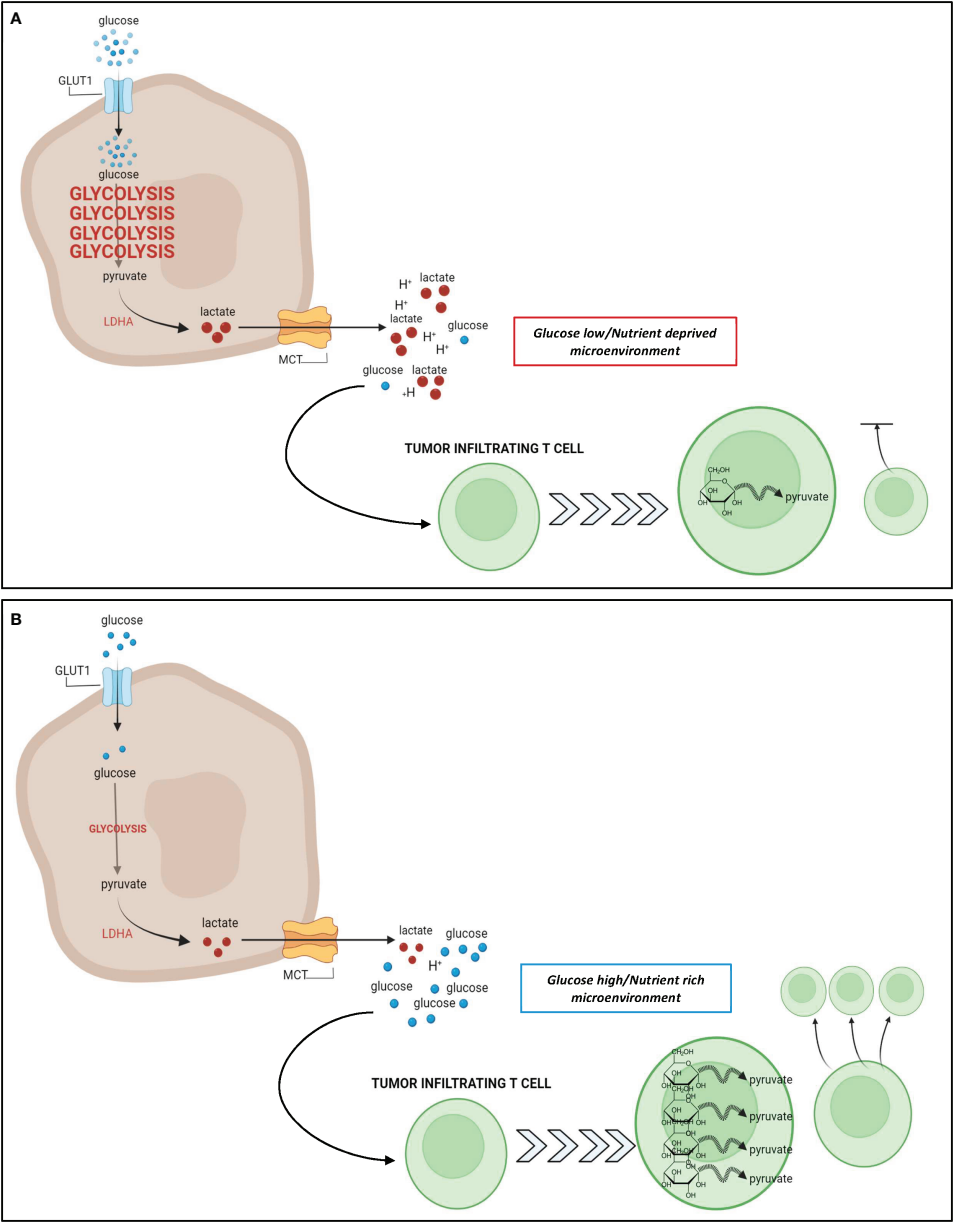
Relationship between tumor glycolysis, glucose abundance, and T cell response. **(A)**: In glycolysis-high tumors such as TNBC, cancer cells take up a large amount of glucose from the extracellular space to fuel their upregulated glycolytic state. The increase in glycolysis generates a large pool of lactate that is excreted from the cancer cell leading to a hostile, acidic, and nutrient deprived TME. Due to the increased glucose uptake by cancer cells, there is a paucity of glucose remaining in the TME for utilization by T cells that infiltrate the tumor. These nutrient deprived infiltrating T cells are unable to upregulate glycolysis as effectively which results in stunted proliferation and impaired cytotoxic potential. **(B)**: In glycolysis-low tumors, the cancer cells do not rely heavily on glycolysis for energy metabolism, and their uptake of glucose from the extracellular space is minimal. As a result, there is a higher abundance of glucose and a lower abundance of lactate within the TME, leading to a nutrient rich microenvironment, whereby infiltrating T cells can freely take up glucose for their energy needs. Metabolic reprogramming of these T cells favors glycolysis, and contributes to an enhanced effector T cell response. Figure created in BioRender.com.

Tumor metabolic adaptation through glycolysis poses particular challenges to infiltrating immune effector cells, that also mainly rely on glucose to proliferate and function. T cells undergo massive metabolic reprogramming upon antigen recognition and proper co-stimulation, involving the acquisition of a highly glycolytic phenotype, which is necessary for T-cell proliferation and effector/lytic function (98, 99). This metabolic switch is substantially hampered in T cells trafficking into highly glycolytic tumors that generate a hostile nutrient-deprived TME, thus limiting effector function in TILs (**Figure 1**). This can in turn contribute to immunotherapy resistance (17, 98, 99).

Accordingly, preclinical studies have demonstrated that tumors with low glycolytic capacity are more responsive to T-cell targeted immunotherapies, including immune checkpoint blockade (17, 100–102); whereas a glycolysis-high tumor state has been associated with a less favorable response to these therapies in patients (18, 19, 103). Numerous studies in patients have reported an inverse correlation between intra-tumor T-cell infiltration and the tumor glycolytic state, which was variably assessed by testing expression of glucose transporters or glycolytic enzymes, and glycolytic gene signatures (100, 104–106). Moreover, accumulating evidence is showing that tumors less reliant on glycolysis tend to better respond to immunotherapy, and the identification of glycolytic gene signatures from tissue biopsies has been associated with inferior prognosis as well as decreased progression-free survival upon retrospective analyses in breast cancer and melanoma (18, 107). Retrospective analyses also showed that elevated serum LDH is associated with worse responses to anti-PD1 treatment in melanoma patients (18, 108–110). These studies raise the question of whether the tumor glycolytic state can predict immunotherapy response, particularly in TNBC, considering the metabolic heterogeneity of TNBC as described above.

**Figure 2:**
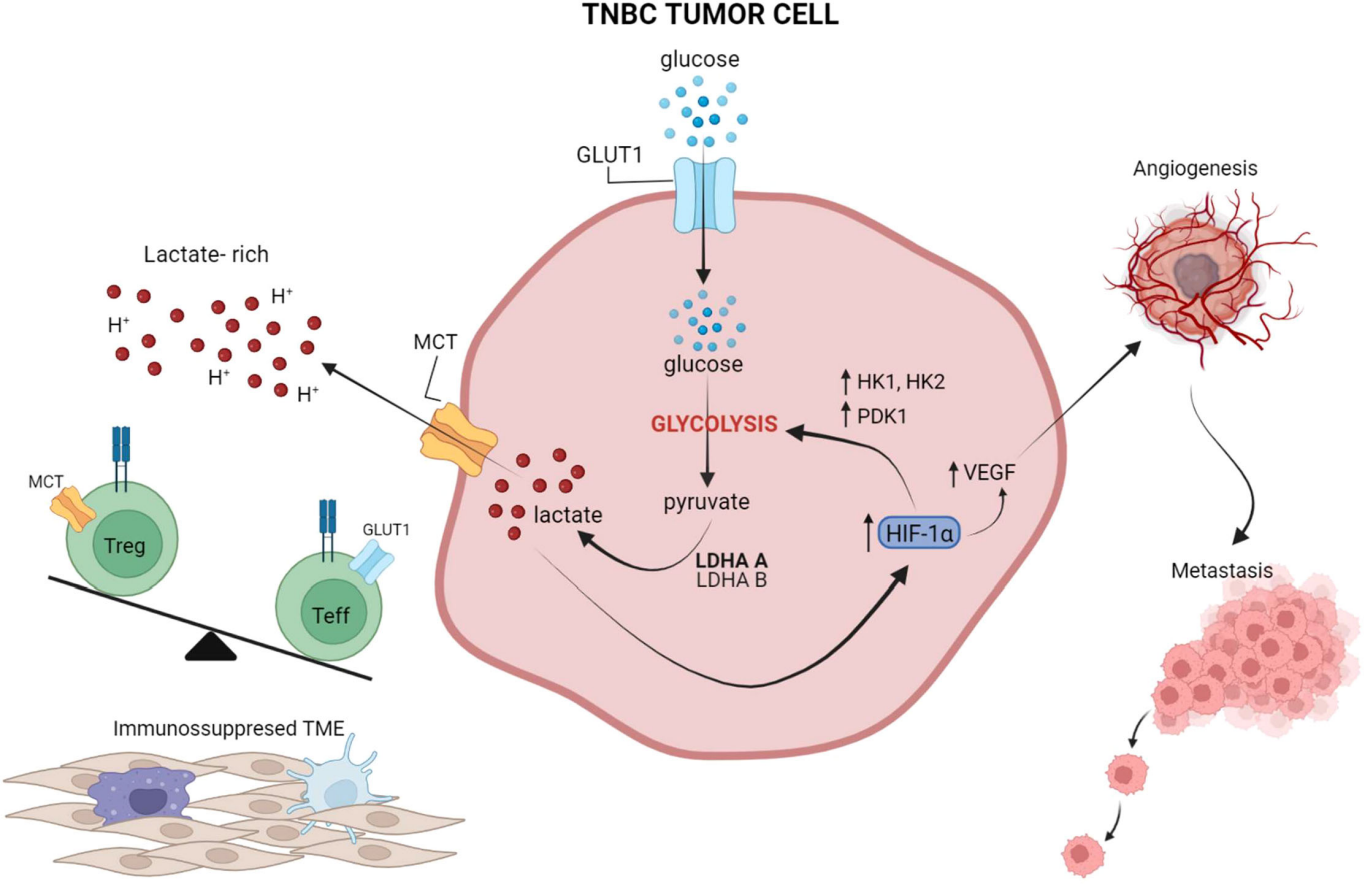
The interplay between tumor glycolysis, the immune microenvironment, and hypoxia. In highly glycolytic TNBC tumors, glucose is rapidly depleted from the microenvironment and lactate is abundantly produced through glycolysis and released into the extracellular microenvironment, leading to an increase in acidity. Lactate also activates HIF-1a which in turn further promotes glucose metabolism through glycolysis, by upregulating HK and PDK1, and induces VEGF production for neo-vessel formation and metastasis dissemination. Preclinical studies suggest that this lactate-rich, glucose-low, and hypoxic environment leads to exclusion of Teff from the TME and favors Treg retention and phenotypic integrity. Overall, these effects reinforce an immunosuppressed microenvironment, which in turn leads to suboptimal responses to ICIs. Figure created in BioRender.com.

A series of preclinical observations have suggested that impairing tumor glycolysis can lead to enhanced immunosurveillance and decreased tumor growth, highlighting an opportunity to target tumor glycolysis as a means of improving the efficacy of immunotherapy (18, 106). In a study by Zappasodi et. al, a mouse TNBC model with knockdown (KD) of lactate dehydrogenase A (LDHA) – a key glycolytic enzyme subunit – showed decreased glycolytic activity and delayed tumor growth in immunocompetent mice (17). When treated with neoadjuvant immune checkpoint blockade using an anti-CTLA-4 antibody, mice bearing LDHA-KD (glycolysis-low) had prolonged survival compared to control tumors. Importantly, upon neoadjuvant CTLA-4 blockade, glycolysis-low tumors displayed greater T-cell infiltration, with more extracellular glucose available for infiltrating Tregs to reprogram their metabolism toward effector-like T-cell cells that produce IFN-γ and TNF-α (17). These data suggest a novel mechanism whereby inhibiting tumor glycolysis can predispose the microenvironment to respond to immune checkpoint blockade by favoring intratumoral T-cell infiltration and Treg inactivation, which may be driven by the glucose:lactate ratio in the TME (17). This study highlighted that increased tumor glycolysis and local lactate leads to greater Treg integrity and immunosuppression (**Figure 2**), whereas lower tumor glycolysis and increased local glucose destabilizes Tregs, and hampers immunosuppressive forces (17). Similarly, Watson et al. showed that intra-tumor Tregs utilize tumor-derived lactate and prefer lactate over glucose as a metabolic substrate in the TME; further, lactate uptake is necessary for Tregs to maintain their suppressive function (101). Unlike effector T cells, Tregs do not rely on glycolysis for their function and can survive in a glucose-low environment, thus explaining their ability to survive well in a hostile lactate-rich TME (101, 111–113). It has also been suggested that increased lactate in the TME can drive Treg polarization from naïve T cells (111). Of note, deleting the lactate transporter MCT1 selectively in Tregs significantly improved the long-term responses to anti-PD-1 in several murine solid tumor models (101).

Interfering with tumor glycolysis to increase the anti-Treg effects of CTLA-4 blockade seems particularly attractive to treat breast cancer, where Tregs are known to play an important role in immune evasion (114). Targeting glycolysis in breast cancer can also alleviate the immune suppressive mechanisms of myeloid derived suppressor cells (MDSCs). In fact, knockdown of LDHA in TNBC mouse models was found to reduce tumor-infiltrating MDSCs in the TME and was associated with decreased tumor burden (103). 

Taken together, these findings indicate that Tregs and tumor cells can establish a metabolic symbiosis which is reinforced in the setting of highly glycolytic tumors and leads to an immunosuppressive TME (**Figure 1**). It will be key to determine the extent to which these mechanisms are reproduced in human tumors and can contribute to immunotherapy resistance, especially in the context of highly glycolytic TNBC. This will be crucial to guide new combination therapies incorporating inhibitors of tumor glycolysis (e.g. LDH inhibitors), lactate transporters (MCT inhibitors), or pathways supporting glycolysis (e.g. PI3K/mTOR; MAPK; c-Myc) to break this symbiosis and potentiate the activity of immunotherapy. Understanding whether preferential targeting of glycolysis in tumors versus immune cells may be achieved by selecting tumors with particularly high glycolytic capacity or by establishing specific administration schedules; this will be fundamental for successful combinations with immunotherapy.

Recent studies have started to investigate how to harness this metabolic information to improve the treatment of TNBC. Gong et al. (19) classified TNBCs into three metabolic subtypes (MPS 1-3) by profiling metabolic gene expression, metabolite abundance, and genomic drivers in 360 human TNBC tumors using multi-omics analyses. The second group, MPS2, comprised 37% of tumors and was designated as the glycolytic subtype due to upregulated carbohydrate metabolism. MPS2 closely correlated with the basal luminal 1 (BL1) subtype described by Lehmann in 2016 and showed an upregulation of glycolytic genes, higher tumor grade, and worse prognosis (5, 19). The combination of an LDH inhibitor (FX-11) plus PD-1 blockade in murine TNBC models representing the 3 metabolic subtypes showed significant improvement in anti-tumor activity selectively against the MPS2 tumor subtype, with an increase in cytolytic CD8+TILs and NK cells was detected upon treatment (19). These studies highlight the potential to harness the metabolic heterogeneity of TNBCs for classification into distinct metabolic subtypes that can be used to predict therapy response and to allocate patients to optimal treatment.

### Relationship between tumor hypoxia, glycolysis, and immunotherapy response

Tumor glycolysis very closely correlates with hypoxia in the TME, which contributes to tumor aggressiveness. Tumor hypoxia is a spatially and temporally heterogeneous phenomenon, influenced by tumor type, tumor volume, specific organ or tissue localization, micro-vessel density, blood flow, oxygen diffusion and consumption rates (115). Pimonidazole is the most commonly used staining for hypoxia and remains the ‘gold standard’ to measure hypoxia in preclinical and clinical studies, with the caveat that it can only be used to assess hypoxia *ex vivo* in tissue. Multiple noninvasive imaging methods measuring oxygen distributions and hypoxia-related factors, such as perfusion, are in development with some being already available (115), and can help elucidate how tumor hypoxia changes with tumor growth and with treatment. These quantitative measures of metabolic changes in the TME over time may constitute important indicators of tumor evolution and response to treatment (116).

The regulatory factor of the hypoxia-signaling pathway in cells is the hypoxia-inducible transcription factor 1 (HIF-1) consisting of an oxygen-regulated α-subunit and a stable β-subunit (117, 118). Another isoform, HIF-2α, also serves an important role in cellular response to hypoxia (119, 120). Cancer cells can acquire HIF-1α somatic mutations leading to increased HIF-1α expression and/or activity (121), which can be a result of both genetic and physical/metabolic alterations within tumors that impact HIF-1 transcriptional activity. HIF proteins are also controlled by several post translational modifications (PTM) affecting protein stability, localization, ability to form protein complexes, and DNA binding, highlighting the importance of tight regulation of this pathway during homeostasis (122). Most HIF-1 up-regulated genes are involved in oxygen sensing and utilization *via* angiogenesis and metabolic reprogramming, through vascular endothelial growth factor (*VEGF*), erythropoietin (*EPO*), and glucose transporters (*GLUT*), as well as key glycolytic enzymes, such as HK1 and HK2. In addition to pathways important for maintaining oxygen homeostasis in tumors, the downstream targets of HIF-1 are involved in autophagy, apoptosis, redox homeostasis, inflammation and immunity, invasion, and metastasis (123). Moreover, hypoxia through HIF-1 downregulates genes involved in DNA repair (124). Moreover, in addition to stimulating glycolytic genes, HIF-1 also represses mitochondrial function and oxygen consumption by inducing pyruvate dehydrogenase kinase 1 (PDK1). PDK1 phosphorylates and prevents pyruvate dehydrogenase from using pyruvate to fuel the mitochondrial TCA cycle, leading to a decreased in mitochondrial oxygen consumption and a relative increase in intracellular oxygen tension (125). 

Notably, the end products of glycolysis, lactate and pyruvate, can promote HIF-1α protein stability and activate HIF-1-inducible gene expression (126), linking glycolysis to activation of hypoxia-downstream signaling pathways independent of oxygen, which reinforces glycolytic metabolism (127). Moreover, lactate has the ability to promote the protein expression of carbonic anhydrase IX (CAIX), a cellular pH regulatory component that has been shown to be more predominantly expressed in TNBC compared with other breast cancer subtypes and associated with inferior survival (128, 129). CAIX is considered an important factor for tumor progression through cell-cell de-adhesion, and stimulation of migration and invasion. Lactate stimulates CAIX expression through HIF-1α stabilization independently of hypoxia, making CAIX a crucial effector of lactate that responds to the metabolic microenvironment and in turn enhances cancer aggressiveness (130). Promising results have been reported in preclinical studies inhibiting CAIX in TNBC tumors. Hedlund et al. targeted CAIX with a small molecule inhibitor, SLC-0111, which led to reduced metastatic burden and decreased primary tumor vascular permeability in mice with orthotopic TNBC xenografts, and these effects were synergistic when SLC-0111 was combined with ICIs (131, 132). Taken together, these data would support the rationale to evaluate the role of CAIX inhibition in combination with immunotherapy in TNBC.

The effect of tumor lactate on regulating hypoxia response may contribute to immune evasion and immunotherapy resistance. As mentioned above, elevated tumor lactate levels and LDH-A expression are associated with worse prognosis in a variety of tumor types, and downregulation of LDH-A can delay metastases development. This suggests that in the microenvironment of glycolysis-low tumors – due to decreased lactate levels – HIF-1α accumulates less efficiently and neovascularization may be limited, which in turn can favor immune cell infiltration and control of tumor growth. Accordingly, analyses of breast cancer databases have shown an association between increased LDH-A and HIF-1α gene expression with poorer outcomes. In addition, patients with poor outcomes that tended to have high LDH-A/HIF-1α levels also showed low CD3E/CD4 and CD8A expression (133). Consistently, reducing glycolysis in highly aggressive breast cancer models by LDHA-KD was found to significant impact the TME, disease evolution, progression, and development of metastases (133–135). Specifically, Serganova et al. showed that mice bearing 4T1 TNBC tumors with LDH-A-KD had reduced HIF-1α expression in the TME along with lower vascularity, and increased tumor immune infiltrate. These changes were associated with inhibition of metastasis formation, prolonged survival and improved responses to immunotherapy (17, 133).

## Discussion

Overall, these findings indicate the importance of evaluating hypoxia in the TME together with tumor glycolysis to obtain more robust indicators of the tumor metabolic state and use them to better predict treatment response and to guide rational immunotherapy-based combinations with increased efficacy. The mechanistic interactions between glycolysis and hypoxia and their impact on the immune microenvironment are particularly relevant in TNBC – a highly aggressive tumor type with strong preferences for glycolysis as a form of energy metabolism. The pre-clinical studies in TNBC described above highlight important metabolic factors that should be explored in the clinical setting for TNBC, in an effort to improve immunotherapy responses by focusing on tumor metabolism and hypoxia.

### Future directions

The aggressive nature of TNBC necessitates efforts to optimize treatment combinations, and the biologic heterogeneity of TNBC justifies precision-medicine therapeutic approaches to achieve this (19). Recent findings about TNBC metabolic reprogramming and its impact on the immune microenvironment have revealed potential new targetable vulnerabilities for counteracting the resistance to immunotherapy seen in certain patients. Preclinical evidence in mouse models and correlation analyses in patients indicate that TNBC tumors can be categorized based on their glycolytic state, and this may offer an opportunity to improve patients’ allocation to treatment (17–19, 136). In addition, the data reviewed in this article suggest that tumor preferences for energy metabolism can be harnessed and reprogrammed to sensitize the TME to immunotherapy responses. Specifically, these studies support the hypothesis that glycolysis-low compared to glycolysis-high tumors are more likely to respond to ICIs and that there is potential to improve immunotherapy efficacy against glycolysis-high tumors by downregulating their glycolytic capacity.

Inhibiting glycolysis within cancer cells is a promising therapeutic strategy that has been studied primarily in the preclinical arena. The main barrier to clinical translation of these agents is the concern for systemic toxicity and targeting the glycolytic inhibition to cancer cells without altering normal cell metabolism. Some clinical studies have begun to evaluate the combined treatment of inhibitors of glycolysis with chemotherapy and radiation. The glycolytic inhibitor 2-deoxy-D-glucose (2-DG), which competitively inhibits glucose transporters, was one of the initial examples studied clinically but its progress had been hindered by systemic toxicity (137). Original studies evaluated its use as a radiosensitizer in cranial neoplasms and showed efficacy in tumor xenografts (138, 139). This drug did reach a phase I/II trial in prostate cancer but was terminated early due to lack of effect (140). Another drug, lonidamine, which inhibits glycolysis through hexokinase II (HK2) inhibition, has been shown to have multiple targets in energy metabolic pathways. It has been studied in a variety of clinical settings and has shown efficacy in delaying resistance to temozolomide and acting as a radiosensitizer in glioblastoma treatment, as well as in potentiating the effects of chemotherapy in solid tumors (140–142). Targeting MCT1 is another therapeutic strategy, which has been explored using the drug AZD3965, a fist in class MCT1 inhibitor. This drug has been studied in a phase I clinical trial in lymphoma with promising results and no significant toxicities (143). More recently, studies have evaluated the implementation of dietary interventions to inhibit glycolysis. In one ongoing trial, a short term ketogenic and low caloric diet during chemotherapy is being investigating in patients with metastatic breast cancer (144).

The above clinical evidence opens the door for a new paradigm in cancer treatments of tumor types which depend heavily on glycolysis for their energy needs. In the realm of TNBC, these targets are exciting prospects, particularly when considering them in combination with ICI’s. Further phase I trials are needed to substantiate the safety of these agents, and it will be imperative to select targets which limit the inhibitory effects on glycolysis in the normal tissue. We look forward to additional clinical studies using these agents, and foresee great promise of glycolytic inhibitors in the treatment of TNCB. 

The immunogenicity of TNBC is intimately linked to its metabolic capacity. Targeting tumor glycolysis is an attractive approach to tailor treatment to the specific tumor biology and microenvironment. The studies discussed in this review underscore the potential to improve long-term clinical benefit by taking advantage of the metabolic heterogeneity of TNBC tumors and selecting patients based on the tumor glycolytic state for optimized therapeutic intervention. This approach may lead to improved responses to immunotherapy in both early-stage and advanced disease settings, which can have significant clinical impact on patients’ long-term survival.

## Author contributions

The first draft of the manuscript was written by AS and all authors commented, reviewed, and edited each version of the manuscript including the tables and figures. AS: Contributed to the inception, writing, reviewing, and editing each version. RZ: Contributed to the inception, writing, reviewing, and editing each version. IS: Contributed to writing, reviewing, and editing each version. KB: Contributed to writing, reviewing, and editing each version. SD: Contributed to writing, reviewing, and editing each version. EA: Contributed to the inception, writing, reviewing, and editing each version. All authors contributed to the article and approved the submitted version.
